# Inheritance and allelism of morphological traits in eastern redbud (*Cercis canadensis* L.)

**DOI:** 10.1038/hortres.2015.49

**Published:** 2015-10-28

**Authors:** David J Roberts, Dennis J Werner, Phillip A Wadl, Robert N Trigiano

**Affiliations:** 1 Department of Horticultural Science, North Carolina State University, Box 7609, Raleigh, NC 27695-7609, USA; 2 Department of Entomology and Plant Pathology, University of Tennessee, 2505 E.J. Chapman Dr. 370 Plant Biotechnology Bldg., Knoxville, TN 37996, USA

## Abstract

Inheritance of purple, gold, and variegated foliage types, weeping architecture, and double flower was explored in F_1_, F_2_, and backcross families resulting from controlled hybridization of eastern redbud (*Cercis canadensis* L.). Potential allelic relationships were explored when possible. Inheritance analysis in families derived from controlled hybridization of ‘Covey’ (green leaf) and ‘Forest Pansy’ (purple leaf) suggest that purple leaf color and weeping architecture are both controlled by single recessive genes, for which the symbols *pl1* and *wp1* are proposed, respectively. Inheritance of gold leaf was explored in families of ‘Covey’ (green leaf) × ‘Hearts of Gold’ (gold leaf). Interpretation of inheritance of gold leaf in these families was confounded by the recovery of a leaf color phenotype in the F_2_ family unlike either parent. However, data suggested the action of a single locus controlling gold leaf color in ‘Hearts of Gold’, and that instability of gold leaf expression may be based on transposable element activity. Segregation of gold leaf in the F_2_ families of ‘Texas White’ [green leaf (*C. canadensis* var. *texensis*)] × ‘JN2’ [gold leaf (The Rising Sun)] did not fit a Mendelian ratio. Analysis of progeny of ‘Silver Cloud’ and ‘Floating Clouds’ (both showing white/green leaf variegation) with non-variegated cultivars demonstrated that variegation in ‘Silver Cloud’ is controlled by a single recessive nuclear gene, while variegation in ‘Floating Clouds’ is controlled by cytoplasmic factors. The symbol *var1* is proposed for the gene controlling variegation in ‘Silver Cloud’. Double flower in progeny derived from ‘Flame’ (double flower) suggested that double flower is dominant to single flower, and that ‘Flame’ is heterozygous at the double-flower locus, for which the symbol *Df1* is proposed. Allelism studies showed that the gene controlling purple leaf in ‘Forest Pansy’ is allelic to the purple leaf gene in ‘Greswan’ and that the gene controlling weeping phenotype in ‘Traveller’ (*C. canadensis* var. *texensis*) is non-allelic to the weeping gene found in ‘Covey’. Allelism of the gold leaf trait in ‘Hearts of Gold’ and ‘JN2’ was investigated, but no clear conclusions regarding allelism could be made due to recovery of leaf color phenotypes unlike either parent.

## Introduction

Eastern redbud (*Cercis canadensis* L.) is a small landscape tree that exhibits considerable morphological diversity, including variation in plant architecture, plant size, and flower and leaf colors. Specific cultivars and botanical varieties found in eastern redbud possess a variety of phenotypic characteristics whose inheritance can be studied and documented through strictly controlled breeding studies.^1^ Determination of inheritance for these traits can help plant breeders better understand the genetic mechanisms that lead to specific phenotypes and allow greater control while manipulating these characteristics in a breeding program. Despite the relatively high number of observable characteristics, no named genes yet exist for any phenotypic variants found in eastern redbud. Furthermore, little is known about modes of inheritance for these desirable traits, or allelic relationships between similar phenotypic variants that have arisen independently in different lineages.

The objectives of this study were to investigate the modes of inheritance for purple, gold, and variegated (green and white) leaf color, weeping architecture, and double flower in *C. canadensis*. We further sought to determine if some of the aforementioned phenotypes found in different cultivars are caused by mutations at the same locus (allelic) or at different loci (non-allelic). Tests for allelism are valuable tools that allow plant breeders to determine if the genes responsible for certain traits are found at the same genetic locus of two or more accessions possessing similar phenotypes. By identifying particular genes as allelic, breeders can better predict how desirable traits will be expressed in future hybridizations.

**Figure 1:**
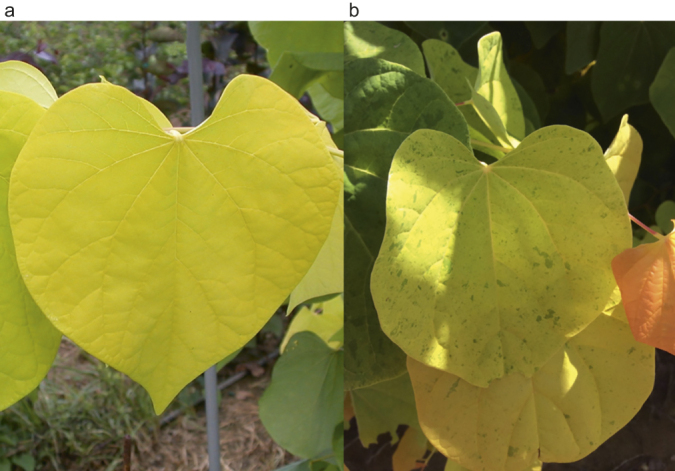
Foliage of *Cercis canadensis* (**a**) ‘Hearts of Gold’ and (**b**) ‘JN2’ showing solid gold color in ‘Hearts of Gold’ and the gold with green spots phenotype of ‘JN2’.

There are several potential allelic relationships in *C. canadensis*. Two purple leaf cultivars of independent origin currently exist: ‘Forest Pansy’ and ‘Greswan’ (Burgundy Hearts). ‘Forest Pansy’ was discovered in 1947 in Tennessee and ‘Burgundy Hearts’ was discovered in the early 2000s in Oklahoma, and both were chance seedlings. Two cultivars of independent origin that show gold leaf color are ‘JN2’ (The Rising Sun) and ‘Hearts of Gold’. Both cultivars have similar but not identical phenotypes. ‘Hearts of Gold’ shows a solid gold leaf, with only slight anthocyanin expression in newly emerging leaves, whereas ‘JN2’ produces a gold leaf with orange overtones in the petiole and newly emerged leaves. As the leaves of ‘JN2’ mature they show numerous small, dark green spots scattered over the golden adaxial leaf surface (Figure 1).

Two weeping cultivars of independent origin were tested for allelism: Covey (Lavender Twist), discovered in New York state in 1991, and Traveller (*C. canadensis* var. *texensis*), discovered in Texas in 1989. The weeping phenotypes vary slightly between cultivars. ‘Covey’ demonstrates an abrupt weeping habit, whereas the weeping habit of ‘Traveller’ is slightly more open and spreading.

In this study, we describe the inheritance and allelism of various phenotypic traits using segregation data obtained from F_1_, F_2_, BC_1P1_, and BC_1P2_ families. Furthermore, linkage among these traits was investigated when possible. Results presented in this manuscript were accumulated over 17 years of breeding efforts aimed at developing improved cultivars of redbud for the landscape and nursery industry.

## Materials and methods

Unless otherwise stated, all controlled hybridizations were performed under greenhouse conditions using potted trees and utilized identical pollination techniques. Because *C. canadensis* is self-incompatible, flowers did not require emasculation. Flowers on the female parent were pollinated using a fine artist’s brush, using pollen obtained from shoots of the male parent forced in the greenhouse. Flowers were pollinated on the female parent when keel petals were fully extended, and separated from the banner and wing petals. Unless otherwise stated, all F_2_ families were obtained by growing F_1_ family trees in isolation at the Sandhills Research Station, Jackson Springs, North Carolina (USA). Isolation blocks were separated by at least 300 m to minimize the potential for cross-contamination with other families. Pollination among F_1_ trees was accomplished by the presence of wild pollinating insects. Crosses, families generated, and progeny numbers are shown in Table 1.

### Inheritance of purple leaf color and weeping architecture

Inheritance of purple leaf color and weeping architecture, and potential linkage between these traits, was investigated in F_1_, F_2_, BC_1P1_, and BC_1P2_ families derived from the controlled hybridization of ‘Covey’ (green leaf, weeping architecture) and ‘Forest Pansy’ (purple leaf, non-weeping architecture). ‘Covey’ has a leaf color that is typical of wild-type *Cercis* ((The Royal Horticultural Society Colour Chart, London, England), RHS green group 137A), and ‘Forest Pansy’ has leaf color RHS purple group N77A. The F_1_ family was created by isolating a potted tree of both ‘Covey’ and ‘Forest Pansy’ within a pollination cage. A nest of bumblebees (*Bombus pennsylvanicus* De Geer) was placed inside the cage to accomplish cross-pollination. The BC_1P1_ and BC_1P2_ families were created by utilizing controlled hybridization techniques previously described. The F_2_ family was created by growing the 23 trees of the F_1_ family in isolation as described above. F_2_ seed was harvested from individual F_1_ trees and kept separate, and F_2_ progeny were later established in the field. Characterization of leaf color was conducted in early summer at a time when purple leaf color is highly expressed. Architecture (weeping vs. non-weeping) was scored on trees in their second growing season to ensure unambiguous characterization of weeping phenotypes. Preliminary studies in our program suggested that weeping growth habit and purple leaf color were each controlled by single recessive genes. Chi-square analysis was used to test for goodness of fit to an expected ratio of 3:1 for leaf color (green leaf: purple leaf) and architecture (non-weeping: weeping), and to test for goodness of fit to an expected 9:3:3:1 ratio for a di-hybrid cross involving both traits. Linkage between the purple leaf and weeping architecture was tested using contingency analysis. Since four different F_2_ families were utilized in this study (each derived from separate full-sib F_1_ trees), data from each family were tested for departure from homogeneity.

### Inheritance of gold leaf color – ‘Hearts of Gold’

Inheritance of the gold leaf trait was explored in F_1_, F_2_, BC_1P1_, and BC_1P2_ families derived from hybridization of ‘Covey’ (green leaf) × ‘Hearts of Gold’ (gold leaf). Families were generated as previously described. Progeny were scored for leaf color in the greenhouse shortly after germination, at the second true leaf stage. We initially hypothesized that F_2_ progeny would segregate into only green leaf and gold leaf categories. However, some individuals showed a leaf color phenotype unlike either parent, classified as ‘bleached’, showing cotyledons and first true leaves that were white to light yellowish-green with sparse green streaking. Hence, segregating progeny were separated into one of three categories based on leaf color (green, gold, and bleached). Since five different F_2_ families were utilized in this study (each derived from separate full-sib F_1_ trees), data from each family were tested for departure from homogeneity.

### Inheritance of gold leaf color – ‘JN2’ (The Rising Sun)

Inheritance of the gold leaf phenotype was further explored in F_1_, F_2_, BC_1P1_, and BC_1P2_ families derived from hybridization of ‘Texas White’ (*C. canadensis* var. *texensis*, green leaf) and ‘JN2’ (gold leaf with small green spots). In all families, ratios were calculated for the segregating families and tested using the chi-square test for goodness of fit for leaf color. For F_2_ analysis, two approaches were undertaken. In the first case, F_2_ seed was harvested from approximately 100 randomly chosen F_1_ trees and bulked (bulked F_2_ family). In the second approach, about 50–75 seeds were harvested from 38 randomly selected F_1_ trees and kept separate for analysis. Progeny from 5 of these 38 separate F_2_ families failed to segregate for both gold leaf color and gold/green (mottled) leaf color. Thirteen of the F_2_ families produced only green and mottled leaf progeny, lacking gold leaf segregants, prompting the authors to investigate whether or not the F_1_ parents of the families not segregating for gold leaf were true hybrids of ‘Texas White’ and ‘JN2’. Simple sequence repeat (SSR) loci were utilized to determine the genetic identity of 12 of the F_1_ parents not segregating for gold leaf color. Leaf tissue was collected from both parental genotypes (‘JN2’ and ‘Texas White’) and 12 selected intra-specific hybrids and stored at −80 °C until genomic DNA isolation. Tissue was homogenized by grinding in liquid nitrogen and DNA isolated using the Qiagen Dneasy Plant DNA isolation kit (Qiagen, Valencia, CA, USA). The manufacturer’s instructions were followed for DNA isolation except that 1.5% polyvinyl polypyrolidone was added to buffer AP1. Total DNA was quantified with the NanoDrop ND-1000 ultraviolet–visible Spectrophotometer (NanoDrop Technologies, Wilmington, DE, USA), DNA quality was determined using 2% agarose gels stained with ethidium bromide and visualized in the 2000 Gel Documentation System (Bio-Rad Laboratories, Hercules, CA, USA).

Primer pairs from *C. canadensis* microsatellite loci^2^ that were polymorphic between ‘JN2’ and ‘Texas White’ were selected and screened against 12 putative hybrids to confirm true hybrid origin. Microsatellite amplification was completed using the following conditions: 10 μL PCR reactions contained 0.4 ng genomic DNA, 2.5 mM MgCl_2_, 1 × GeneAmp PCR Buffer II (Applied Biosystems, Foster City, CA, USA), 0.2 mM dNTPs, 0.25 µM primer, 0.6 U AmpliTaq Gold DNA polymerase (Applied Biosystems), and sterile, nanopure water. Cycling conditions were as follows: 1 cycle of 94 °C for 5 min, 35 cycles of 94 °C for 40 s, 55 °C for 40 s, 72 °C for 30 s, and 1 cycle of 72 °C for 4 min. PCR products were sized on the QIAxcel Capillary Electrophoresis System (Qiagen, Valencia, CA, USA) using an internal 25-bp DNA step ladder.

Raw allele length data for each sample and locus were binned into allelic classes using the program FLEXIBIN.^3^ We utilized a conservative 2 bp allelic class size range because of the 2 bp resolution of the QIAxcel Capillary Electrophoresis System (Qiagen). All loci selected for hybrid confirmation were polymorphic and differed by at least 4 bp in the parents to eliminate potential resolution issue during allele separation. The multilocus genotypic data for each hybrid was compared to the parents for hybrid confirmation. To simplify hybrid confirmation, only loci that were homozygous in each respective parent were utilized. Thus, every hybrid should have two alleles detected for each locus analyzed.

### Inheritance of double flower

**Figure 2:**
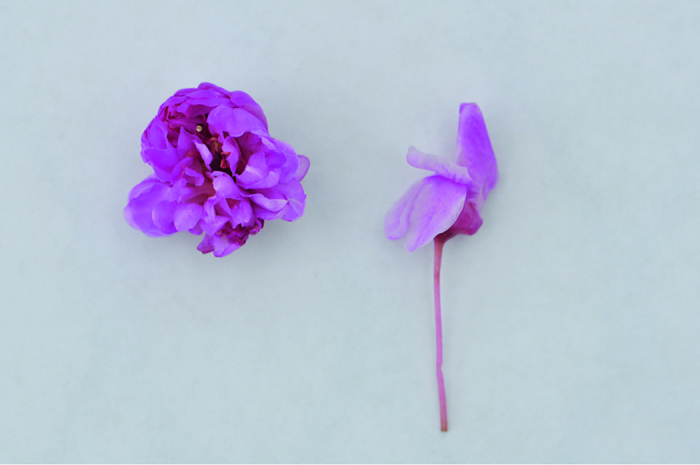
Double (left) vs. single flower of *Cercis canadensis*.

Inheritance of the double flower phenotype (Figure 2) was investigated in F_1_ progeny derived from controlled hybridization of ‘Dwarf Alba’ (single flower) × ‘Flame’ (double flower). The double-flowered ‘Flame’ is essentially female sterile, and rarely sets fruit. The double-flowered F_1_ progeny recovered from the aforementioned hybridization were also female sterile, precluding the recovery of F_2_ progeny. Compared to the typical five petals in a flower of redbud, ‘Flame’ typically shows 30–40 petals per flower (unpublished data). Atypically, in 2005 a tree of ‘Flame’ growing among a diverse collection of redbud cultivars set fruit. Hence, viable seed was collected and obtained from Hidden Hollow Nursery (Belvidere, TN, USA) and open-pollinated seedlings were evaluated to obtain additional evidence on inheritance of double flower.

### Inheritance of variegated leaf

Inheritance of the variegated leaf phenotype was investigated in five different F_1_ families generated from the hybridization of ‘Floating Clouds’ (variegated leaf) with non-variegated cultivars ‘Covey’ (cross made reciprocally), NC2006-14, ‘Texas White,’ and ‘JN2’. Inheritance of variegated leaf was investigated further in F_1_ and F_2_ families derived from reciprocal hybridizations of ‘Silver Cloud’ (variegated leaf) and ‘Covey’. In both cases, ratios were calculated for the segregating families and tested using the chi-square test for goodness of fit. Both ‘Floating Clouds’ and ‘Silver Cloud’ have variegated green leaves with white sectors. However, both were discovered as chance seedlings of independent origin. Their phenotypes are similar, but ‘Floating Clouds’ has more prominent white sectors and leaves are more resistant to sunscald (personal observation).

### Allelism of purple leaf phenotypes

Preliminary analysis suggested that purple leaf color in ‘Forest Pansy’ is controlled by a single recessive gene. In order to determine if the genes controlling purple leaf color in ‘Forest Pansy’ and ‘Greswan’ are allelic, controlled hybridizations were accomplished under greenhouse conditions. Because both ‘Forest Pansy’ and ‘Greswan’ show a high degree of female sterility, ‘Greswan’ was utilized as the male parent, and the purple leaf cultivar ‘Ruby Falls’ (female fertile), derived from ‘Forest Pansy’ was used as the female parent. If controlled by the same locus, and if both cultivars are homozygous for the recessive mutation, one would predict all F_1_ progeny to exhibit purple leaf color. Conversely, if the purple phenotype is controlled by different loci in the two cultivars, one would expect all F_1_ progeny to exhibit green leaf color. Progeny were scored for purple leaf color in the greenhouse, after development of two true leaves. Additionally, open-pollinated seed of ‘Greswan’ was obtained from trees growing at the JC Raulston Arboretum (Raleigh, NC, USA) and from Green Leaf Nursery, (Park Hill, OK, USA) to verify if the purple leaf phenotype exhibited by ‘Greswan’ is recessive. Because eastern redbud is self-incompatible, all open-pollinated seed originate from outcrossing. Open-pollinated seedlings should show green leaves if the purple leaf phenotype is recessive in ‘Greswan’, assuming no outcrossing to purple leaf plants.

### Allelism of weeping phenotypes

Allelism of the genes controlling weeping phenotype in ‘Covey’ and ‘Traveller’ was investigated. Because ‘Traveller’ shows almost absolute male and female sterility, it cannot be used in controlled crosses. However, a rare open-pollinated seedling of ‘Traveller’ (designated NC2011-1) was obtained in our program in 2011. The seedling is non-weeping, but presumably heterozygous for the weeping allele. Hence, hybridization between NC2011-1 and ‘Ruby Falls’ (derived from ‘Covey’) is informative for allelism determination, and reciprocal crosses were made in the greenhouse. If the weeping phenotypes are controlled by allelic genes, one would predict recovery of 50% weeping progeny in the F_1_ family. Alternatively, if the weeping genes are non-allelic, one would expect only non-weeping offspring in the F_1_ family. Initial scoring of progeny was performed approximately 7 months after germination, at the end of the first growing season. Subsequently, all progeny were again scored in mid-summer of the second growing season to confirm accuracy of scoring.

### Allelism of gold leaf phenotypes

Both gold leaf cultivars (Hearts of Gold and JN2) were crossed in reciprocal combinations to investigate allelism of the genes controlling gold leaf color. Controlled hybridizations were made in a greenhouse, and progeny were scored for leaf color in the first growing season in both a greenhouse and field setting. If controlled by the same locus, and if both cultivars are homozygous for the recessive mutation, one would predict all F_1_ progeny to exhibit gold leaf color. Conversely, if the gold leaf phenotype is controlled by different loci in the two cultivars, one would expect all F_1_ progeny to exhibit green leaf color.

## Results and discussion

### Inheritance of purple leaf color and weeping architecture

**Figure 3:**
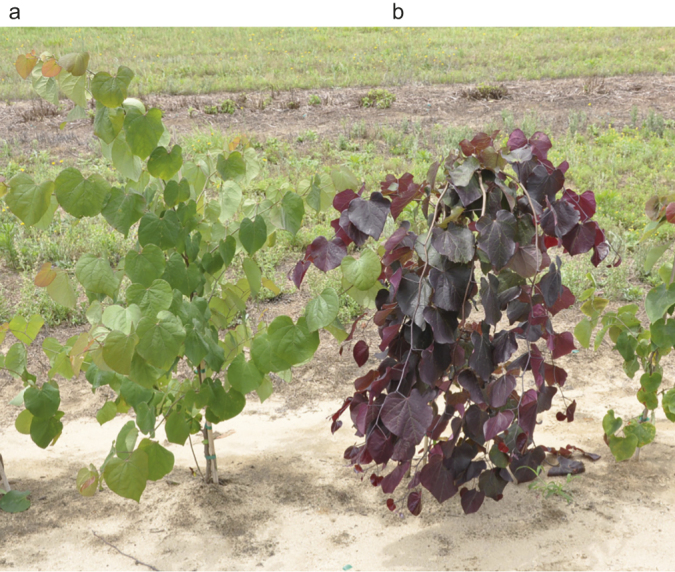
*Cercis canadensis* ‘Covey’ × ‘Forest Pansy’ F_2_ trees exhibiting segregation for: (**a**) green leaf, non-weeping phenotype, and (**b**) purple leaf, weeping phenotype.

**Figure 4:**
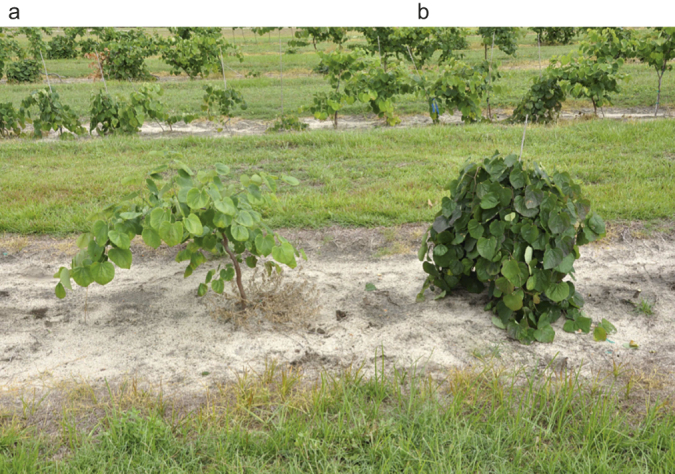
*Cercis canadensis* ‘Covey’ × ‘Forest Pansy’ segregating progeny exhibiting: (**a**) semi-pendulous habit (scored as non-weeping) and (**b**) weeping habit.

The F_1_ family of ‘Covey’ × ‘Forest Pansy’ consisted of 23 plants, all showing non-weeping growth habit and green leaves. The F_2_ seed was derived from random intercrossing of 23 F_1_ trees and collected from four separate F_1_ trees, providing 1586 progeny. Leaf color of F_2_ progeny could be easily discerned in early stages of development, typically by observing the first true leaves. Cotyledons of purple leaf plants were green, identical to the cotyledons of green leaf progeny. Segregating F_2_ progeny exhibiting the purple leaf phenotype was clearly discernable from those exhibiting the green phenotype in early summer, the time of scoring in the field (Figure 3). Characterization of the weeping phenotype in F_2_ progeny proved to be more difficult than the purple leaf phenotype in early stages of development. Scoring progeny in the second growing season was considerably easier than doing so in year one. Numerous progeny, particularly in the first growing season, exhibited an intermediate phenotype between weeping and non-weeping. Assuming single gene action, these progeny possibly represent heterozygotes for the weeping gene. As a result, only those individuals that clearly demonstrated the rigid and distinct weeping phenotype as shown by ‘Covey’ (Figure 4) were categorized as weeping.

A chi-square test for heterogeneity among the four F_2_ families for segregation of both purple leaf color and weeping architecture was non-significant (*P* = 0.09 and *P* = 0.87, respectively), so data were combined prior to analysis. Segregation for leaf color fit the expected ratio of 3:1 (green leaf: purple leaf) at *P* = 0.02 (Table 2). A slight underrepresentation of purple leaf progeny accounted for the minor distortion in the F_2_ data. The F_1_ and F_2_ data indicate that purple leaf is recessive to green leaf, and that purple leaf is controlled by a single recessive gene. This conclusion is supported by backcross data (Table 2), showing a lack of purple leaf segregants in the BC_1P1_ family. BC_1P2_ progeny derived from the backcross of (‘Covey’ × ‘Forest Pansy’) × ‘Forest Pansy’ segregated in the expected ratio of 1:1 (green leaf: purple leaf) at *P* = 0.82 (Table 2). We propose that purple leaf color in ‘Forest Pansy’ is controlled by a single recessive gene, designated *pl1*. Furthermore, we also propose that ‘Forest Pansy’ has a genotype *pl1pl1* and that those plants exhibiting the wild-type green leaf have a genotype *Pl1Pl1*.

Analysis of combined data for the four F_2_ families showed segregation for the weeping phenotype fit a ratio of 3:1 (non-weeping: weeping) at *P* = 0.26 (Table 3). Although all F_1_ plants were classified as non-weeping, they did demonstrate a slight semi-pendulous growth habit in their first 2 years of growth, subsequently less distinct as trees aged. The F_1_ and F_2_ data indicate that weeping architecture is recessive to non-weeping, and weeping is controlled by a single recessive gene for which we propose the designation *wp1*. We propose that ‘Covey’ has a genotype of *wp1wp1*. Our hypothesis that weeping is recessive to non-weeping was further supported through BC_1P1_ segregants which fit the expected ratio of 1:1 (non-weeping: weeping) at *P* = 0.44 (Table 3) and BC_1P2_ segregants which showed a lack of weeping progeny. Results of contingency analysis suggest that the genes responsible for purple leaf color and weeping architecture are not linked (*P* = 0.69, Table 4). Additionally, combined co-segregation analysis for the purple leaf and weeping traits fit a ratio of 9:3:3:1 (*P* = 0.05, Table 5) that would be predicted for a di-hybrid cross involving two recessive genes. This further supports lack of linkage between the purple leaf and weeping loci.

### Inheritance of gold leaf color – ‘Hearts of Gold’

**Figure 5:**
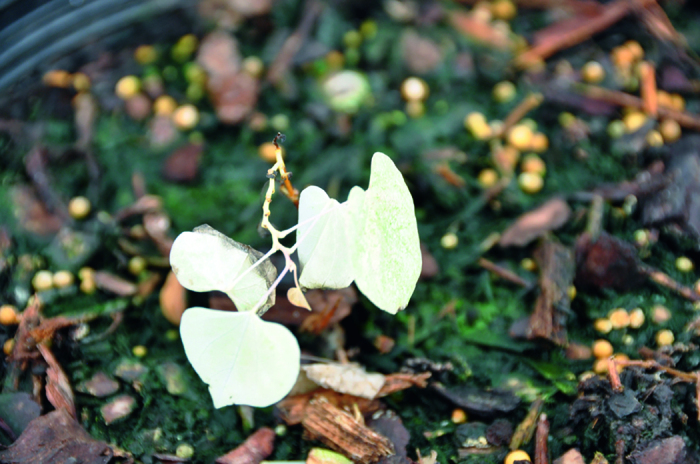
Bleached progeny derived from *Cercis canadensis* ‘Covey’ × ‘Hearts of Gold’, approximately 1 month old and demonstrating chlorophyll deficiency. The majority of bleached seedlings died.

**Figure 6:**
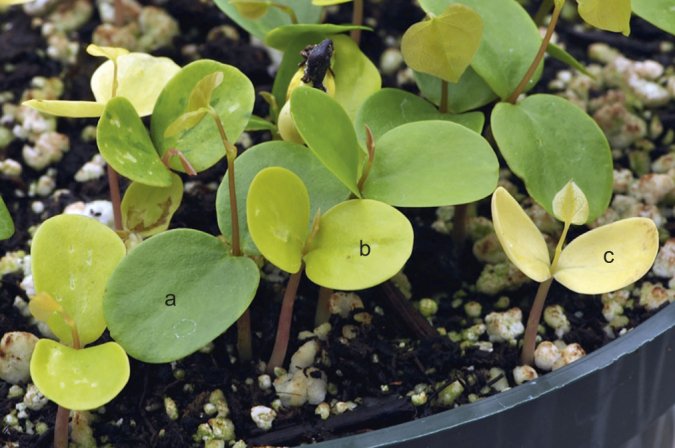
Examples of progeny phenotypes derived from *Cercis canadensis* ‘Covey’ × ‘Hearts of Gold’ segregating for cotyledon color, classified as: (**a**) green cotyledons, (**b**) gold cotyledons, and (**c**) bleached cotyledons.

The F_1_ family derived from ‘Covey’ × ‘Hearts of Gold’ consisted of 37 plants, all non-weeping with green leaves, expected if the gold leaf trait is simply inherited and recessive. The 37 F_1_ trees were grown in isolation and were randomly intercrossed by natural pollinators, and F_2_ seed was harvested from five F_1_ trees and kept separate. We initially predicted that F_2_ progeny would segregate into only the parental green leaf and gold leaf categories. However some segregants demonstrated a leaf color phenotype unlike either parent, classified as bleached. Bleached segregants produced small, distorted leaves, nearly albino, but with sparse light yellow spots (Figure 5). Typically, bleached progeny did not survive past the seedling stage. The F_2_ and backcross plants were separated into one of three categories based on leaf phenotype (green, gold, and bleached), which was assessed at the second true leaf stage. Cotyledon color was highly predictive of leaf color. Green leaf plants expressed a cotyledon color typical of wild type (Royal Horticultural Society (RHS) green group 137A; Figure 6). Plants scored as gold leaf had cotyledons that were distinguishably lighter in color (RHS yellow-green group 144A) than the green group. The bleached category possessed cotyledons that were very pale yellow (RHS greyed-yellow group 160D). Those bleached progeny that survived in the greenhouse had very light yellow leaves that became necrotic upon exposure to full sun.

**Figure 7:**
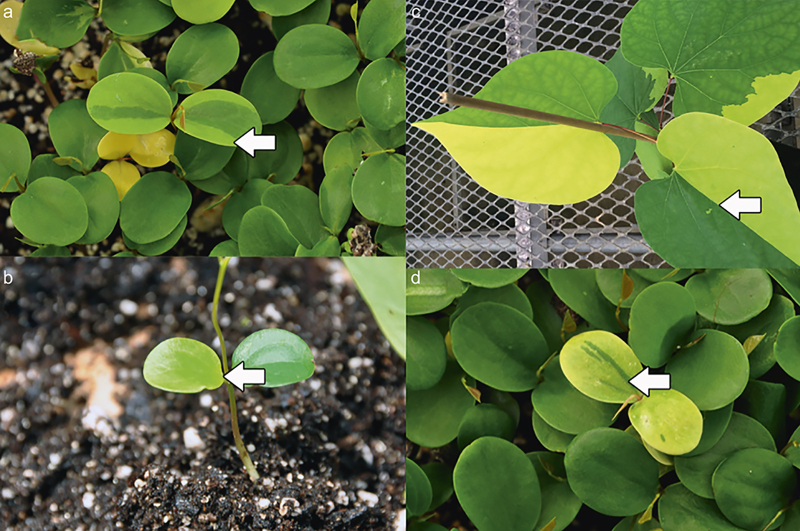
Progeny derived from *Cercis canadensis* hybridizations involving ‘Hearts of Gold’, demonstrating putative transposon-mediated variegation. (**a**) ‘Covey’ × ‘Hearts of Gold’ F_2_ showing a green cotyledon with gold variegation. (**b**) ‘Covey’ × ‘Hearts of Gold’ F_2_ showing both a green and gold cotyledon. (**c**) ‘Covey’ × ‘Hearts of Gold’ F_2_, 4 months old showing green-gold leaf variegation on opposite sides of the midrib. (**d**) ‘Covey’ × ‘Hearts of Gold’ F_1_ showing gold leaf cotyledon with green variegation. Arrows indicate variegated sectors.

The segregation in the F_2_ of green, gold, and bleached progeny did not match any known Mendelian segregation ratio, perhaps suggesting the interaction of more than one locus. However, when all non-green segregants were combined into a single category (gold + bleached), each of the five F_2_ families fit a ratio of 3:1 (green leaf: gold + bleached leaf; Table 6). A chi-square test for heterogeneity among the five F_2_ families was conducted, using combined data for gold and bleached as a single phenotypic category. The heterogeneity chi-square was non-significant (*P* = 0.55), so data were combined prior to analysis. Combined data fit a ratio of 3:1 (green leaf: gold + bleached leaf) at *P* = 0.06 (Table 6). Demonstration of a 3:1 ratio (green leaf: gold + bleached leaf) after combination of gold and bleached leaf phenotypes is suggestive of the action of only one locus controlling both the gold leaf and bleached phenotypes. Although this segregation ratio is more consistent with inheritance involving a single gene recessive trait, it does not explain the recovery of numerous bleached progeny. One possible explanation is transposable element (TE) activity at the locus controlling gold leaf, leading to a small subset of progeny producing a very low amount of chlorophyll (bleached phenotype). Insertion of non-autonomous DNA-based active rice transposon one (nDart1) has resulted in pale yellow variegation in rice, and mutants of *Arabidopsis thaliana* that underwent *Ds* insertion events have resulted in albino phenotypes.^4,5^ Similarly, *Ds* insertion mutants of *Arabidopsis* experienced a disruption of ribosome release factor 1, which proved to be critical in chloroplast development and PSII activity.^6^ The observed ratio of 3:1 (green leaf: gold + bleached leaf) coupled with the presence of limited chlorophyll in the cotyledons of bleached progeny suggests that the bleached phenotype may be gold leaf segregants that experienced a TE insertion or excision event early in development, rendering them highly disabled in chlorophyll synthesis. Several instances in our study were documented in which a viable bleached segregant ultimately developed into a very light gold phenotype, reflecting the transient nature of TE-based phenotypes. TE activity could be the cause for the moderate degree of phenotypic instability witnessed in some F_2_ progeny derived from ‘Covey’ × ‘Hearts of Gold’ (Figure 7).

The BC_1P1_ family showed only green leaf progeny, as predicted if gold leaf is recessive. Segregants from the BC_1P2_ family fell into the same three phenotypic categories found in the F_2_ (green, gold, and bleached). Even when gold and bleached categories were combined into a single group (gold + bleached), this family showed a deficiency of non-green leaf progeny that resulted in a distortion of the expected 1:1 ratio (green leaf: gold + bleached leaf), at *P* < 0.001 (Table 6). The basis for this distortion cannot be explained. These results confirm that the gold leaf trait in ‘Hearts of Gold’ is not cytoplasmic, as ‘Hearts of Gold’ was used as the male parent in the initial controlled hybridization that created the F_1_ family. Additionally, our data show that progeny derived from ‘Hearts of Gold’ exhibited a moderate degree of phenotypic instability, more so than progeny derived from the other gold leaf cultivar, JN2.

### Inheritance of gold leaf color – ‘JN2’ (The Rising Sun)

**Figure 8:**
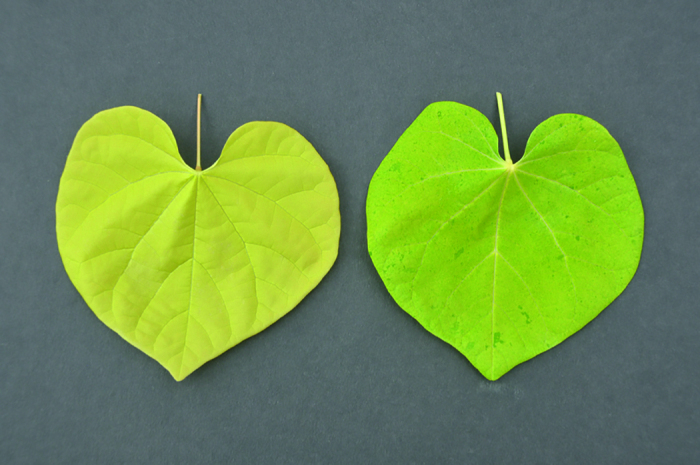
Comparison of solid gold leaf phenotype and mottled leaf phenotype recovered in the F_2_ of *Cercis canadensis* ‘Texas White’ × ‘JN2’.

The F_1_ family of ‘Texas White’ (green leaf) × ‘JN2’ (gold leaf with green spots) consisted of 463 progeny, all of which showed green leaf color. The F_1_ progeny were intermated (natural pollinators), and F_2_ seed was collected from about 100 F_1_ trees and bulked. The bulk F_2_ family segregated for both green, entire gold (no green spots), and mottled leaf phenotypes (Figure 8). The mottled leaf phenotype was similar to gold leaf, but showed an overlay of small, mottled green sectors, similar to the phenotype typically expressed on leaves of ‘JN2’, the gold leaf parent. Considerable variation existed among the mottled phenotype plants, ranging from subtle to pronounced. No bleached progeny were recovered among F_2_ segregants in the ‘Texas White’ × ‘JN2’ family, unlike the ‘Covey’ × ‘Hearts of Gold’ F_2_ family, in which bleached progeny were recovered in moderate numbers. Cotyledon color was highly predictive of leaf color in segregating families. Green leaf plants expressed a cotyledon color typical of wild type (RHS green group 137A). Plants scored as gold leaf had cotyledons that were distinguishably lighter in color (RHS yellow-green group 144A). The mottled category possessed cotyledons that were identical to the gold cotyledon classification. The F_2_ and backcross plants were separated into one of three categories based on leaf phenotype (green, gold, and mottled), which was assessed at the second true leaf stage. Segregation in the F_2_ of green, gold, and mottled progeny did not match any known Mendelian segregation ratio, perhaps suggesting the interaction of more than one locus. As with the ‘Covey’ × ‘Hearts of Gold’ study, all non-green segregants were pooled into a single category (gold + mottled) and analyzed for a goodness of fit to a 3:1 ratio (green leaf: gold + mottled leaf). However, analysis of the bulk F_2_ family did not fit the expected ratio of 3:1 (green leaf: gold + mottled leaf) at *P* < 0.001, showing a highly significant underrepresentation of the gold + mottled progeny category (Table 7).

Subsequently, in order to better characterize the segregation distortion in the bulk F_2_ family, a second experiment was conducted. For this experiment, additional F_2_ seed was collected from 38 individual F_1_ trees, and kept separate for analysis. An average of 55 F_2_ progeny were grown on from each of these individual F_1_ trees to further assess leaf color segregation. Consistent with the results of the bulked F_2_ family, green, gold, and mottled progeny were recovered. There was a significant deviation (*P* = 0.05) in 24 of the 38 families from the expected 3:1 ratio (green leaf: gold + mottled leaf), again with a deficiency in ‘gold + mottled’ progeny (Table 8). Gold leaf F_2_ segregants in these families exhibited uniform gold leaf color, more similar to ‘Hearts of Gold’ and distinct from the mottled gold phenotype of ‘JN2’. Unexpectedly, 5 of the 38 F_2_ families (10, 28, 31, 33, 38) failed to show any gold leaf or mottled segregants (303 total segregating progeny). Based on a single gene model (with gold leaf being recessive to green leaf), the likelihood of recovering only green leaf segregants from a population of 303 segregating progeny is 1.39 × 10^−38^. Thirteen additional F_2_ families (8, 9, 13, 14, 15, 29, 30, 32, 34, 36, 39, 49, 50) showed no gold progeny, but only green and mottled progeny.

All progeny derived from the BC_1P1_ family (‘Texas White’ × (‘Texas White’ × ‘JN2’)) were green leaf (Table 7), supporting the hypothesis that gold leaf is recessive to green leaf. The BC_1P2_ family (‘JN2’ × (‘Texas White’ × ‘JN2’)) segregated for both green and gold leaf, but the mottled phenotype was not recovered. Segregation in the BC_1P2_ family deviated significantly from the expected test ratio of 1:1 (green leaf: gold leaf) (*P* = 0.007), showing a deficiency of gold leaf segregants (Table 7). Results from backcross families are suggestive of a single recessive gene controlling gold leaf in ‘JN2’, but the highly significant underrepresentation of gold leaf and mottled progeny recovered in the F_2_ and BC_1P2_ families is difficult to explain. In this particular BC_1P2,_ the heterozygous F_1_ was used as the male parent. It is possible that pollen carrying the gold leaf allele does not effectively compete with pollen carrying the green leaf allele, resulting in a deficiency of gold leaf segregants. A similar phenomenon has been observed in *Pisum sativum*, where pollen carrying the stringless allele grows more slowly than wild-type pollen, resulting in a deficiency of stringless progeny in F_2_ and certain backcross families.^7^ However, this explanation does not adequately explain the total absence of gold leaf and mottled segregants in five of the sampled F_2_ families.

Alternatively, the distortion in segregation ratios in the bulked F_2_ family, and the lack of gold leaf segregants in 5 of the 38 individual F_2_ families could be explained if contamination occurred during pollination to create the F_1_ family, resulting in non-hybrid progeny being sampled. To rule out this possibility, the five F_1_ trees that failed to segregate for gold leaf or mottled progeny were analyzed along with seven additional F_1_ trees that segregated only for green leaf and mottled progeny. Six microsatellite loci (386b, 508a, 658a, 671a, 732a, 830a) were used to confirm hybridization between the parents ‘JN2’ and ‘Texas White’. These loci were homozygous and polymorphic between the parents. All of the putative hybrids (NCTWRS-9, NCTWRS-10, NCTWRS-14, NCTWRS-28, NCTWRS-31, NCTWRS-32, NCTWRS-33, NCTWRS-34, NCTWRS-36, NCTWRS-38, NCTWRS-39, and NCTWRS-50) tested were heterozygous and had a single allele from each parent, thus confirming hybridization between ‘Texas White’ and ‘JN2’

Since all tested F_1_ trees proved to be true hybrids, one would predict them to be heterozygous for the gold leaf allele derived from ‘JN2’. That five of these families failed to segregate for gold or mottled leaf color, and 13 additional families (seven verified as true hybrids using SSR’s) segregated only for mottled but not gold progeny suggests that some factor is impacting transmission of the gold leaf allele. Activity of TE could be responsible for the distortion in the observed ratios and complete lack of gold leaf and mottled progeny in specific F_2_ families. If the gold leaf mutation is based on a TE insertion, a TE excision event during male gametogenesis could restore function and result in a modified allele that functionally behaves as a green leaf allele, resulting in F_1_ progeny that are homozygous for green leaf. These homozygous green F_1_ progeny could account for the underrepresentation of gold leaf and mottled segregants in the bulk F_2_ family, and lack of gold and mottled segregation in F_2_ families derived from individual F_1_’s.

The leaf phenotype of the ‘JN2’ parent, showing a predominantly gold leaf with numerous random islands of green of varying size, is suggestive of TE activity. The mottled leaf phenotype recovered in progeny of ‘JN2’ could be the result of a partial TE excision at the gold allele during gametogenesis or early embryo development, resulting in partial restoration of function and creation of a unique leaf color phenotype similar to ‘JN2’, and unlike the green leaf ‘Texas White’ parent. Recovery of solid gold progeny lacking green spots in F_2_ family may also represent TE element-based variation. A similar phenomenon may explain the bleached phenotype in the ‘Covey’ × ‘Hearts of Gold’ F_2_ progeny.

Assuming maternal inheritance of chloroplasts and mitochondria in redbud, our results confirm that the gold leaf trait in ‘JN2’ is not inherited cytoplasmically as ‘JN2’ was used as the male parent in the initial controlled hybridization that created the F_1_ family.

### Inheritance of double flower

Two F_1_ progeny derived from the hybridization of ‘Dwarf Alba’ × ‘Flame’ were both double flowered, suggesting dominance of double flower. Analysis of F_1_ progeny derived from open pollination of ‘Flame’ showed segregation for single flower and double flower. Segregation for single and double flower did not fit the expected test ratio of 1:1 (single flower: double flower) at *P* < 0.001 (Table 9), showing a deficiency of single-flowered progeny. However, the data suggest that double flower is controlled by a single dominant gene, for which we propose the designation *Df1,* and that ‘Flame’ is heterozygous at this locus.

### Inheritance of variegated leaf – ‘Floating Clouds’ and ‘Silver Cloud’

Five F_1_ families generated from hybridizations involving ‘Floating Clouds’ (variegated leaf) showed that leaf variegation in that cultivar is inherited cytoplasmically (Table 10). In the four instances where ‘Floating Clouds’ was utilized as the female parent, all F_1_ progeny exhibited variegated foliage. In the one instance in which ‘Floating Clouds’ was the male parent in a hybridization with ‘Covey’, all F_1_ progeny exhibited green foliage.

Analysis of the F_1_ and F_2_ families derived from the hybridization of ‘Covey’ × ‘Silver Cloud’ shows that variegation in ‘Silver Cloud’ is controlled by a nuclear gene, with the two F_1_ progeny both showing green leaves. These F_1_ progeny were intermated, and seed was harvested off of each tree separately yielding two F_2_ families. Segregation analysis of the F_2_ families (NC99-18-1 and NC99-18-2) fit the expected test ratio of 3:1 (green leaf: variegated leaf) at *P* = 0.02 and *P* = 0.74, respectively (Table 11). These findings indicate that variegation from ‘Silver Cloud’ is controlled by a single recessive gene for which we propose the designation *var1.* Hence, the genotype of ‘Silver Cloud’ is *var1var1.*

### Allelism of purple leaf phenotype

F_1_ progeny derived from hybridization of ‘Ruby Falls’ × ‘Greswan’ all showed purple leaf color, except for a single individual, which was green leaf. Progeny derived from open pollination of ‘Greswan’ in a field setting were green leaf, with the exception of three purple leaf individuals (Table 12). Because eastern redbud is self-incompatible, all open-pollinated seedlings derived from open pollination of ‘Greswan’ are half-sibs, confirming that the purple leaf trait in ‘Greswan’ is recessive. Hence, a preponderance of purple offspring derived from the allelism test hybridization is not caused by dominance of purple leaf color in ‘Greswan’. The presence of three purple leaf progeny in the open-pollinated family derived from ‘Greswan’ could be explained by outcrossing to another purple leaf cultivar. These results strongly support that the genes controlling purple leaf color in ‘Forest Pansy’ and ‘Greswan’ are allelic. We propose that genes conferring purple leaf color in ‘Greswan’ be designated *pl1pl1* in accordance with the previous designation established for ‘Forest Pansy’.

### Allelism of weeping architecture

Progeny derived from reciprocal hybridizations between NC2011-1 (non-weeping and heterozygous for the weeping gene derived from ‘Traveller’) and ‘Ruby Falls’ (homozygous for the weeping gene derived from ‘Covey’) were all non-weeping except for a single weeping individual, recovered in the family derived from NC2011-1 as the female parent. This individual is presumably the result of a rare self-pollination event in NC2011-1. A total of 316 progeny were evaluated in the reciprocal families combined. Recovery of 50% weeping progeny would have been expected if the genes were allelic, hence these results show the genes are non-allelic. Alternatively, it is possible that the gene that confers weeping in ‘Traveller’ is dominant and that ‘Traveller’ is heterozygous at that locus. If this were the case, NC2011-1 may possess the recessive (non-weeping) form of the allele, which would render all F_1_ progeny derived from hybridization with ‘Ruby Falls’ non-weeping as well. However, the recovery of a weeping individual from the cross using NC2011-1 as the female parent argues against this possibility. Because the weeping phenotypes in these two cultivars differ slightly, the potential exists that unique weeping forms will be recovered in the F_2_ generation, based on selection of progeny expressing weeping genes from both parents.

### Allelism of gold leaf phenotype

**Figure 9:**
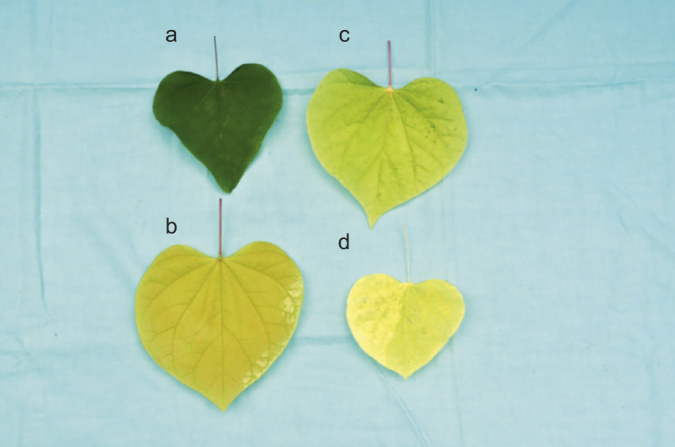
Examples of phenotypic categories established for mature leaves in progeny derived from the hybridization of *Cercis canadensis* ‘Hearts of Gold’ × ‘JN2’ and the reciprocal: (**a**) wild-type green, (**b**) gold, (**c**) chartreuse, and (**d**) bleached.

Reciprocal crosses were made between gold leaf cultivars Hearts of Gold (entire gold) and JN2 (gold with green spots) to test for allelism of the gold leaf trait. We expected to recover either all green leaf (genes non-allelic) or all gold leaf (genes allelic) progeny from these crosses, but both green and gold leaf progeny were recovered, contradictory to a simple affirmation or rejection of allelism. In addition, two other leaf color phenotypes were observed. The bleached phenotype, described earlier in the manuscript, was recovered, as well as a fourth category, designated as chartreuse. Plants classified as chartreuse exhibited a cotyledon color that was intermediate between the gold and bleached categories. Chartreuse plants showed very light gold color in mature leaves (RHS greyed-yellow group 160A). Interestingly, cotyledon color varied considerably among segregating offspring, and appeared to be highly associated with leaf color, hence progeny were initially placed into the four phenotypic categories (green, gold, bleached, and chartreuse) based on cotyledon color. Plants were then grown on for 2–4 months and scored again based on leaf color (Figure 9), so that the accuracy of scoring leaf color phenotypes at the cotyledon stage could be assessed. In the case of both families, the most frequently occurring phenotypes fell into the gold and green leaf categories.

The inherent difficulty of accurately scoring progeny at the cotyledon growth stage is illustrated by the change in frequency of progeny in each category upon final scoring of leaf color (Table 13). The majority of ‘Hearts of Gold’ × ‘JN2’ F_1_ progeny originally designated in the cotyledon stage as green maintained this phenotype; however, a small percentage was later determined to belong to either the gold or chartreuse phenotypes. A small percentage of the progeny scored initially as gold were later determined to belong to either the green or chartreuse categories.

The majority of progeny classified initially in the cotyledon stage as chartreuse developed into plants with very light gold-colored leaves and an airbrushed overtone of very small, numerous, light green spots, similar to but distinct from the ‘mottled’ phenotype of ‘JN2’. Of the bleached progeny derived from ‘JN2’ × ‘Hearts of Gold’, 56% proved to be seedling-lethal. The remaining bleached progeny that survived past the seedling stage in the greenhouse ultimately developed into plants later reclassified as chartreuse. Upon placement in a field setting, all chartreuse segregants showed significant leaf burn and perished. These results suggest that plants classified as chartreuse may simply have a different degree of expression of the bleached phenotype. A moderate number of progeny in this study exhibited considerable phenotypic instability, demonstrating green and gold sectoring on cotyledons and/or leaves of individual plants (Figure 7). This instability was observed in all phenotypic categories.

Progeny that were generated through reciprocal crosses of these two gold leaf parents did not conform to ratios that would be expected from simple allelic or non-allelic inheritance patterns. Consistent with families generated for the gold leaf inheritance study derived from ‘Hearts of Gold’, reciprocal crosses of ‘Hearts of Gold’ and ‘JN2’ yielded not only green leaf and gold leaf progeny, but bleached and chartreuse progeny as well (Figure 9). One would have expected all gold leaf progeny if the genes were allelic, or conversely, all green leaf if the genes were non-allelic. The recovery of multiple leaf color phenotypes ranging from near albino, to light and bright gold, to green, and the observed phenotypic instability for leaf color in individual plants is highly suggestive of instability conferred by TE activity in the gold leaf locus. It is possible, given the relatively high number of gold leaf segregants, that genes coding for gold leaf color in ‘Hearts of Gold’ and ‘JN2’ are indeed allelic, but transposon-mediated events during gametogenesis or early embryo development impacted phenotypic expression, leading to the observed phenotypes and instability of expression. Both ‘Hearts of Gold’ and ‘JN2’ demonstrate classic signs of transposon activity. As previously mentioned, ‘JN2’ exhibits small islands of green scattered across a gold leaf surface, reminiscent of the ‘broken colors’ variegation found in the flowers of *Mirabilis jalapa* L., a species known for transposon-mediated variegation.^8^ The mutagenic nature of transposons can be suppressed by small interfering RNA’s, especially in gametes that could transmit transposed elements to the next generation. Also, both mobility and expression of TEs can be transiently activated in the vegetative nuclei (tube nuclei) of pollen, which can ultimately lead to new somatic transpositions.^9^ The bleached, green, and chartreuse phenotypes recovered from hybridizations involving ‘Hearts of Gold’ and ‘JN2’ could be the result of such transient activation. TE activity in pollen of ‘Hearts of Gold’ or ‘JN2’ could cause unpredictable transmission of the gold leaf phenotype in different genetic backgrounds. It is possible the bleached and chartreuse progeny recovered in these families were gold leaf segregants whose phenotypes were each altered by TE activity in a different way.

## Conclusions

The modes of inheritance for several phenotypic mutations in *Cercis canadensis* have been documented. The purple leaf phenotype is controlled by a single recessive gene (*pl1pl1*) in ‘Forest Pansy’ allelic to the purple leaf gene in ‘Greswan’. Weeping architecture in ‘Covey’ is controlled by a single recessive gene (*wp1wp1*), non-allelic to the mutation conferring weeping architecture in ‘Traveller’. Inheritance of the variegated leaf trait in ‘Silver Cloud’ and ‘Floating Clouds’ revealed that variegation in ‘Silver Cloud’ is controlled by a single recessive nuclear gene (*var1var1*), while variegation in ‘Floating Clouds’ is controlled by cytoplasmic factors. The double-flowered phenotype of ‘Flame’ is controlled by a single dominant gene (*Df1Df1*). Inheritance of the gold leaf phenotype found in ‘Hearts of Gold’ and ‘JN2’ is less well understood but likely the result of one major recessive gene. The gold leaf phenotype in both ‘Hearts of Gold’ and ‘JN2’ was transmitted both paternally and maternally. The genes controlling gold leaf in these two cultivars are likely allelic, but this could not be definitively determined. Phenotypic instability in leaf color observed in F_2_ progeny derived from ‘Hearts of Gold’, and from F_1_ progeny derived from ‘Hearts of Gold’ × ‘JN2’ is highly suggestive of TE activity, which could explain the segregation distortion and range of phenotypic variation in leaf color revealed in hybridizations involving ‘Hearts of Gold’ and ‘JN2’. It is possible that the bleached, mottled, and chartreuse phenotypes recovered in families derived from these two cultivars represent variable expression of the gold leaf phenotype altered by TE activity and genetic background.

## Figures and Tables

**Table 1:** *Cercis canadensis* crosses and progeny numbers screened for inheritance of purple leaf color, weeping architecture, gold leaf color, double flower, and variegated leaf.

Cross	Inheritance of trait(s)	Families generated (no. of progeny)^z^
‘Covey’ × ‘Forest Pansy’	Purple leaf/weeping	F_1_(23), F_2_(1586)
‘Covey’ × (‘Covey’ × ‘Forest Pansy’)	Purple leaf/weeping	BC_1P1_(112)
(‘Covey’ × ‘Forest Pansy’) × ‘Forest Pansy’	Purple leaf/weeping	BC_1P2_(318)
‘Covey’ × ‘Hearts of Gold’	Gold leaf	F_1_(37), F_2_(1116)
‘Covey’ × (‘Covey’ × ‘Hearts of Gold’)	Gold leaf	BC_1P1_(123)
‘Hearts of Gold’ × (‘Covey’ × ‘Hearts of Gold’)	Gold leaf	BC_1P2_(349)
‘JN2’ × ‘Texas White’	Gold leaf	F_1_(8)
‘Texas White’ × ‘JN2’	Gold leaf	F_1_(463), F_2_ bulk(1195), F_2_(2,216)
‘Texas White’ × (‘Texas White’ × ‘JN2’)	Gold leaf	BC_1P1_(877)
‘JN2’ × (‘Texas White’ × ‘JN2’)	Gold leaf	BC_1P2_(108)
‘Dwarf Alba’ × ‘Flame’	Double flower	F_1_(2)
‘Flame’ (open pollinated)	Double flower	OP(260)
‘Covey’ × ‘Floating Clouds’	Variegated leaf	F_1_(41)
‘Floating Clouds’ × ‘Covey’	Variegated leaf	F_1_(5)
‘Floating Clouds’ × ‘Texas White’	Variegated leaf	F_1_(127)
‘Floating Clouds’ × ‘JN2’	Variegated leaf	F_1_(61)
‘Floating Clouds’ × NC2006-14	Variegated leaf	F_1_(15)
‘Covey’ × ‘Silver Cloud’	Variegated leaf	F_1_(2), F_2_(1771)
‘Ruby Falls’ × ‘Burgundy Hearts’	Purple leaf allelism	F_1_(196)
‘Burgundy Hearts’ (open pollinated)	Purple leaf allelism	OP(91)
NC2011-1 × ‘Ruby Falls’	Weeping allelism	F_1_(117)
‘Ruby Falls’ × NC2011-1	Weeping allelism	F_1_(199)
‘Hearts of Gold’ × ‘JN2’	Gold leaf allelism	F_1_(47)
‘JN2’ × ‘Hearts of Gold’	Gold leaf allelism	F_1_(266)

z F_2_ plants derived from separate F_1_ trees, except where noted.

**Table 2:** Segregation ratios and goodness of fit for leaf color in F_1_, F_2_, and backcross families derived from hybridization of *Cercis canadensis* ‘Covey’ × ‘Forest Pansy’^Z^.

		Progeny phenotype				
Cross	Family	Green leaf	Purple leaf	Test ratio^Y^	*χ*^2^ (1 df)	*P* value
‘Covey’ × ‘Forest Pansy’	F_1_	23	0	All green		
‘Covey’ × ‘Forest Pansy’	F_2_	1230	356	3:1	5.51	0.02
(‘Covey’ × ‘Forest Pansy’) × ‘Covey’	BC_1P1_	384	0	All green		
(‘Covey’ × ‘Forest Pansy’) × ‘Forest Pansy’	BC_1P2_	162	156	1:1	0.05	0.82

Z Data from four different F_2_ families combined for analysis based on test for heterogeneity (*P* = 0.09).

Y Expected segregation based on a one gene model with purple leaf recessive to green leaf.

**Table 3:** Segregation ratios and goodness of fit for weeping architecture in F_1_, F_2_, and backcross families derived from hybridization of ‘Covey’ × ‘Forest Pansy’^Z^.

		Progeny phenotype				
Cross	Family	Non-weeping	Weeping	Test ratio^Y^	*χ*^2^ (1 df)	*P* value
‘Covey’ × ‘Forest Pansy’	F_1_	23	0	All non-weeping		
‘Covey’ × ‘Forest Pansy’	F_2_	1209	377	3:1	1.52	0.26
‘Covey’ × (‘Covey’ × ‘Forest Pansy’)	BC_1P1_	60	52	1:1	0.59	0.44
(‘Covey’ × ‘Forest Pansy’) × ‘Forest Pansy’	BC_1P2_	318	0	All non-weeping		

Z Data from four different F_2_ families combined for analysis based on test for heterogeneity (*P* = 0.87).

Y Expected segregation based on a one gene model with weeping habit recessive to non-weeping growth habit.

**Table 4:** Contingency analysis to test for linkage between genes for purple leaf color and weeping architecture in F_2_ families derived from hybridization of *Cercis canadensis* ‘Covey’ × ‘Forest Pansy’^Z^.

Phenotype	Observed	Expected	*χ*^2^ (3 df)	*P* value
Green, Non-weeping	929	937.62	0.08	
Green, Weeping	301	292.38	0.25	
Purple, Non-weeping	280	271.38	0.27	
Purple, Weeping	76	84.62	0.88	
Total	1586	1586	1.49	0.69

Z Data from four different F_2_ families combined for analysis based on test for heterogeneity (*P* = 0.63).

**Table 5:** Segregation ratio and goodness of fit to a di-hybrid ratio for weeping and purple leaf traits in the combined F_2_ family derived from hybridization of *Cercis canadensis* ‘Covey’ × ‘Forest Pansy’^Z^.

	Progeny phenotype						
‘Covey’ × ‘Forest Pansy’	Non-weeping, Green leaf	Weeping, Green leaf	Non-weeping, Purple leaf	Weeping, Purple leaf	Test ratio^Y^	*χ*^2^ (3 df)	*P* value
F_2_ combined:	929	301	280	76	9:3:3:1	7.97	0.05

Z Data from four different F_2_ families combined for analysis based on test for heterogeneity (*P* = 0.63).

Y Expected segregation based on a two gene model with purple leaf recessive to green leaf and weeping habit recessive to non-weeping habit.

**Table 6:** Segregation ratios and goodness of fit for individual F_2_ families, combined F_2_ families, and backcross progeny derived from hybridization of *Cercis canadensis* ‘Covey’ × ‘Hearts of Gold’^Z^.

		Progeny phenotype						
Cross	Family	Green	Gold	Bleached	Gold + bleached	Test ratio^X^	*χ*^2^ (1 df)	*P* value
‘Covey’ × ‘Hearts of Gold’	F_1_	37	0	0	0	All green		
‘Covey’ × ‘Hearts of Gold’	F_2_ #2	101	37	0	37	3:1	0.24	0.62
‘Covey’ × ‘Hearts of Gold’	F_2_ #3	130	30	12	42	3:1	0.03	0.86
‘Covey’ × ‘Hearts of Gold’	F_2_ #9	335	71	27	98	3:1	1.29	0.26
‘Covey’ × ‘Hearts of Gold’	F_2_ #12	105	24	2	26	3:1	1.85	0.17
‘Covey’ × ‘Hearts of Gold’	F_2_ #14	193	45	4	49	3:1	2.19	0.09
F_2_ combined^Y^:	F_2_	864	207	45	252	3:1	3.48	0.06
‘Covey’ × (‘Covey’ × ‘Hearts of Gold’)	BC_1P1_	152	0	0	0	All green		
‘Hearts of Gold’ × (‘Covey’ × ‘Hearts of Gold’)	BC_1P2_	252	74	23	97	1:1	68.84	<0.001

Z Progeny phenotype based on leaf color, determined after approximately 30 days of growth under greenhouse conditions.

Y Data combined based on test for heterogeneity (*P* = 0.55).

X Expected segregation based on a one gene model with (gold + bleached) recessive to green. Testing for a 3:1 ratio and 1:1 ratio (green: gold + bleached).

**Table 7:** Segregation ratios and goodness of fit for leaf color in F_1_, F_2_ bulk, and backcross families derived from hybridization of *Cercis canadensis* ‘Texas White’ × ‘JN2’^Z^.

		Progeny phenotype						
Cross	Family	Green	Gold	Mottled	Gold + mottled	Test ratio^Y^	*χ*^2^ (1df)	*P* value
‘Texas White’ × ‘JN2’	F_1_	463	0	0	0	All green		
‘Texas White’ × ‘JN2’ (bulk)	F_2_	1025	85	85	170	3:1	73.98	<0.001
‘Texas White’ × (‘Texas White’ × ‘JN2’)	BC_1P1_	877	0	0	0	All green		
‘JN2’ × (‘Texas White’ × ‘JN2’)	BC_1P2_	68	23	17	40	1:1	7.26	0.007
‘JN2’ × ‘Texas White’	F_1_	8	0	0	0	All green		

Z Progeny phenotype based on leaf color, determined at the two-lead stage, after approximately 30 days of growth under greenhouse conditions.

Y Expected segregation based on a one gene model with (gold + mottled) recessive to green. Testing for a 3:1 F_2_ ratio (green: gold + mottled) and a 1:1 backcross ratio (green: gold + mottled).

**Table 8:** Segregation ratios and goodness of fit for leaf color in 38 individual F_2_ families derived from hybridization of *Cercis canadensis* ‘Texas White’ × ‘JN2’^Z^.

	Progeny phenotype						
F_2_ family #	Green	Gold	Mottled	Gold + mottled	Test ratio^Y^	*χ*^2^ (1 df)	*P* value
1	39	1	1	2	3:1	8.85	0.003
3	43	5	3	8	3:1	2.36	0.12
5	61	5	5	10	3:1	4.51	0.03
6	48	9	5	14	3:1	0.19	0.66
7	76	7	4	11	3:1	7.08	0.01
8	61	0	5	5	3:1	10.69	0.001
9	56	0	2	2	3:1	14.37	0.0002
10	83	0	0	0	3:1	27.67	<0.0001
11	42	8	9	17	3:1	0.46	0.5
12	54	9	2	11	3:1	2.26	0.13
13	40	0	5	5	3:1	4.63	0.03
14	41	0	10	10	3:1	0.79	0.37
15	40	0	5	5	3:1	4.63	0.03
16	60	15	0	15	3:1	1.00	0.32
17	64	11	3	14	3:1	2.07	0.15
18	38	2	2	4	3:1	5.37	0.02
19	81	21	1	22	3:1	0.73	0.39
20	31	10	0	10	3:1	0.01	0.92
21	44	1	3	4	3:1	14.67	0.02
22	54	3	0	3	3:1	11.84	0.0006
24	37	3	1	4	3:1	5.08	0.02
25	29	2	3	5	3:1	1.92	0.17
28	25	0	0	0	3:1	8.33	0.0039
29	55	0	4	4	3:1	10.45	0.0012
30	58	0	3	3	3:1	13.12	0.0003
31	62	0	0	0	3:1	20.67	<0.0001
32	43	0	10	10	3:1	1.06	0.30
33	59	0	0	0	3:1	19.67	<0.0001
34	71	0	4	4	3:1	15.47	<0.0001
36	52	0	5	5	3:1	8.01	0.0047
37	49	1	6	7	3:1	4.67	0.03
38	63	0	0	0	3:1	21.0	<0.0001
39	38	0	3	3	3:1	6.84	0.01
40	28	4	5	9	3:1	0.01	0.92
41	21	2	3	5	3:1	0.46	0.5
46	69	9	3	12	3:1	4.48	0.03
49	23	0	2	2	3:1	3.85	0.05
50	32	0	3	3	3:1	5.04	0.02

Z Progeny phenotype based on leaf color, determined after approximately 30 days of growth under greenhouse conditions.

Y Expected segregation based on a one gene model with (gold + mottled) recessive to green.

**Table 9:** Segregation ratios and goodness of fit for double flower in F_1_ and F_2_ progeny derived from hybridization and open pollination involving *Cercis canadensis* ‘Flame’ (double flower).

		Progeny phenotype				
Cross	Family	Double flower	Single flower	Test ratio^Z^	*χ*^2^ (1 df)	*P* value
‘Dwarf Alba’ × ‘Flame’	F_1_	2	0	all double		
‘Flame’ (open pollinated)	OP	170	90	1:1	24.62	<0.001

Z Expected segregation based on a one gene model with double flower dominant to single flower.

**Table 10:** Segregation ratios for leaf variegation in F_1_ families derived from hybridizations of *Cercis canadensis* ‘Floating Clouds’ with four other parents.

		Progeny phenotype	
Cross	Family	Variegated	Green
‘Covey’ × ‘Floating Clouds’	F_1_	0	41
‘Floating Clouds’ × ‘Covey’	F_1_	5	0
‘Floating Clouds’ × ‘Texas White’	F_1_	127	0
‘Floating Clouds’ × ‘Rising Sun’	F_1_	61	0
‘Floating Clouds’ × NC2006-14	F_1_	15	0

**Table 11:** Segregation ratios and goodness of fit for leaf variegation in progeny derived from hybridizations of *Cercis canadensis* ‘Covey’ × ‘Silver Cloud’.

		Progeny phenotype				
Cross	Family	Green	Variegated	Test ratio^Z^	*χ*^2^ (1 df)	*P* value
‘Covey’ × ‘Silver Cloud’	F_1_	2	0	All green		
‘Covey’ × ‘Silver Cloud’ (99-18-1)	F_2_	1259	365	3:1	5.52	0.02
‘Covey’ × ‘Silver Cloud’ (99-18-2)	F_2_	112	35	3:1	0.11	0.74

Z Expected segregation based on a one gene model with variegated recessive to green.

**Table 12:** Progeny phenotypes derived from hybridization of *Cercis canadensis* ‘Ruby Falls’ × ‘Greswan’, and open pollination of ‘Greswan’.

		Progeny phenotype	
Cross	Family	Purple	Green
‘Greswan’	OP	3	88
‘Ruby Falls’ × ‘Greswan’	F_1_	195	1

**Table 13:** Progeny phenotypes derived from reciprocal crosses of *Cercis canadensis* ‘Hearts of Gold’ and ‘JN2’, characterized by cotyledon color and leaf color^Z,Y^.

Phenotypes	‘Hearts of Gold’ × ‘JN2’	‘JN2’ × ‘Hearts of Gold’
Green	(11) 8	(48) 61
Gold	(19) 16	(100) 87
Chartreuse^X^	(15) 19	(68) 102
Bleached^W^	(16) 4	(88) 16
Total progeny^V^	(61) 47	(304) 266

Z Values within parentheses indicate number of progeny based on cotyledon color.

Y Values to the right of parentheses indicate final number of progeny based on leaf color, determined after about 90 days of growth under greenhouse conditions.

X Values at 3 months are greater than those originally recorded in the cotyledon stage due to bleached progeny developing into the chartreuse phenotype.

W Values at 3 months are lower than those originally recorded at the cotyledon stage due to lethality of bleached seedlings and reclassification of some bleached seedlings as chartreuse.

V Values for total progeny, in parentheses, are less than those originally recorded due to lethality of bleached and chartreuse progeny.
